# Lipid Peroxidation Assessment in Preclinical Alzheimer Disease Diagnosis

**DOI:** 10.3390/antiox10071043

**Published:** 2021-06-29

**Authors:** Carmen Peña-Bautista, Lourdes Álvarez-Sánchez, Inés Ferrer, Marina López-Nogueroles, Antonio José Cañada-Martínez, Camille Oger, Jean-Marie Galano, Thierry Durand, Miguel Baquero, Consuelo Cháfer-Pericás

**Affiliations:** 1 Alzheimer’s Disease Research Group, Health Research Institute La Fe, 46026 Valencia, Spain; carpebau93@gmail.com (C.P.-B.); lourdes_alvarez@iislafe.es (L.Á.-S.); ines_ferrer@iislafe.es (I.F.); miquelbaquero@gmail.com (M.B.); 2 Division of Neurology, University and Polytechnic Hospital La Fe, 46026 Valencia, Spain; 3 Analytical Unit Platform, Health Research Institute La Fe, 46026 Valencia, Spain; marina_lopez@iislafe.es; 4 Data Science Unit, Health Research Institute La Fe (IIS La Fe), 46026 Valencia, Spain; bioestadistica@iislafe.es; 5 Institut des Biomolécules Max Mousseron, IBMM, University of Montpellier, CNRS, ENSCM, 34093 Montpellier, France; camille.oger@umontpellier.fr (C.O.); jeangalano@yahoo.co.uk (J.-M.G.); thierry.durand@umontpellier.fr (T.D.)

**Keywords:** Alzheimer disease, plasma, biomarker, lipid peroxidation, mass spectrometry, preclinical

## Abstract

Alzheimer disease (AD) is an increasingly common neurodegenerative disease, especially in countries with aging populations. Its diagnosis is complex and is usually carried out in advanced stages of the disease. In addition, lipids and oxidative stress have been related to AD since the earliest stages. A diagnosis in the initial or preclinical stages of the disease could help in a more effective action of the treatments. Isoprostanoid biomarkers were determined in plasma samples from preclinical AD participants (*n* = 12) and healthy controls (*n* = 31) by chromatography and mass spectrometry (UPLC-MS/MS). Participants were accurately classified according to cerebrospinal fluid (CSF) biomarkers and neuropsychological examination. Isoprostanoid levels did not show differences between groups. However, some of them correlated with CSF biomarkers (t-tau, p-tau) and with cognitive decline. In addition, a panel including 10 biomarkers showed an area under curve (AUC) of 0.96 (0.903–1) and a validation AUC of 0.90 in preclinical AD prediction. Plasma isoprostanoids could be useful biomarkers in preclinical diagnosis for AD. However, these results would require a further validation with an external cohort.

## 1. Introduction

Alzheimer disease (AD), the most prevalent cause of dementia, is characterized in terms of histopathology by its histological markers. Specifically, an intracellular accumulation of phosphorylated tau (p-tau) protein leads to the formation of neurofibrillary tangles, while an extracellular accumulation of β-amyloid peptide leads to the formation of senile plaques. These markers lead to a synapse loss that causes neuron dysfunction and neurodegeneration. Additionally, tau accumulation is a mechanism shared with other neurodegenerative diseases, while β-amyloid accumulation is supposed to be specific for AD [1]. These histopathological findings are present in the brain before AD symptoms appear. In fact, impairment in amyloid β-42 (Aβ42), and tau proteins in cerebrospinal fluid (CSF) samples are detectable some years before clinical symptoms appear. In this sense, preclinical AD could be defined as biomarker evidence of AD’s pathological changes in cognitively healthy individuals [2]; evidence that can be obtained from cerebrospinal fluid (CSF) biomarkers and amyloid brain Positron Emission Tomography (PET) scan. Therefore, positivity in amyloid brain status identifies preclinical AD in asymptomatic cases. Research focusing on this preclinical AD stage is required in order to advance in the knowledge of AD physiopathological mechanisms, as well as to identify new, early and minimally invasive AD biomarkers, which could be determined in the general population, providing data for a better individual prognosis, new therapeutic targets or other benefits.

Blood samples (plasma, serum) are a promising matrix for identifying potential AD biomarkers [3]. A recent study focused on plasma samples from preclinical AD patients, determining different proteins and peptides (p-tau 181, amyloid-β40, amyloid-β42), showed some evidence that plasma analysis could guide the selection of candidates to receive a diagnosis of their amyloid status, and so reduce the number of amyloid PET scans required to identify amyloid-β-positive individuals [4]. Similarly, Janelidze et al. found that impaired plasma p-tau 217 levels correlated with positivity in the brain before tau-PET in AD cases [5]. In the same way, Suárez-Calvet et al. observed that plasma p-tau 181 was significantly increased in the preclinical stage [6], showing early changes in neuronal tau metabolism. Another study focused on the relationship between plasma amyloid-β and cognitive decline in preclinical AD, revealed specific associations with the decline in episodic memory and executive function [7].

Reviewing non-specific AD biomarkers related to other aspects of preclinical AD, plasma lipocalin-2 was associated with some impairment of executive function, at least in preclinical AD [8]. Additionally, some lipids were identified as potential plasma biomarkers [9]. Moreover, exosomes are an emerging sample matrix [10]. In fact, a recent work showed an early neuronal lysosomal dysfunction [11]. Nevertheless, no conclusive results have been obtained, especially in relation to differential AD diagnosis, as well as in longitudinal studies evaluating clinical progression [12].

Regarding potential physiopathological mechanisms involved in early AD, an increasing number of studies highlight the involvement of oxidative stress, determining several parameters such as oxidatively damaged lipids, proteins and nucleic acids [13,14]. Specifically, lipid peroxidation plays an important role since brain is a susceptible organ characterized by both high lipid content and oxygen consumption. Thus, lipid peroxidation is an important factor in the development of neurodegenerative diseases, especially involving ferroptosis and mitochondrial dysfunction as pathological mechanisms [15]. In this sense, the impairment of lipid peroxidation biomarkers in the brain was found together with histological lesions produced in neurodegenerative diseases, such as brain β-amyloid plaques. In addition, previous studies observed an AD relationship with impaired levels of some plasma lipid peroxidation compounds [16]. It could be explained by the high permeability of the blood-brain barrier since early AD stages [17]. However, a previous study in CSF samples did not show correlations between plasma and CSF samples for any of the studied lipid peroxidation compounds (isoprostanes, neuroprostanes…); also, lipid peroxidation biomarkers in CSF samples did not show significant differences between participant groups [18]. In general, previous studies found that some plasma isoprostanes and neuroprostanes isomers could be useful, to some extent, in clinical or research fields as their levels are different between early symptomatic AD stages (patients with mild cognitive impairment (MCI) due to AD) and healthy controls [19,20].

The aim of the present work is to evaluate the possibility of using these lipid peroxidation compounds as minimally invasive biomarkers of preclinical AD, as well as to evaluate whether its use could be beneficial in the development of a potential prevention approach to be applied to the general asymptomatic population.

## 2. Materials and Methods

### 2.1. Study Design and Participants

In this study involving people with unimpaired cognition, participants with detected preclinical AD (*n* = 12) and a control group with elderly participants without AD pathology (*n* = 31) were included. The preclinical AD group included participants with positive CSF AD biomarkers (β-amyloid-42 < 725 pg·mL^−1^, total tau (t-tau > 485 pg·mL^−1^), phosphorylated tau (p-tau > 56 pg·mL^−1^)), and normal cognitive evaluation test scores (clinical dementia rating (CDR) ≤ 0.5 [21], mini-mental state examination (MMSE) ≥ 27 [22], repeatable battery for the assessment of neuropsychological status delayed memory domain (RBANS.DM) ≥ 85 [23]). The control group included participants with negative levels for CSF AD biomarkers (β-amyloid-42 > 725 pg·mL^−1^, t-tau < 485 pg·mL^−1^, p-tau < 56 pg·mL^−1^) and normal cognitive tests (CDR ≤ 0.5 [21], MMSE ≥ 27 [22], RBANS.DM ≥ 85 [23]) (see Table 1). Participants with major brain disorders, traumatic brain injuries and psychiatric disorders were excluded, as well as participants that were not able to complete the neuropsychological evaluations.

The Ethics Committee from Health Research Institute La Fe (Valencia) approved the protocol (ethical protocol code: 2019/0105) and all included participants signed the informed consent before the study procedures.

### 2.2. Sample Collection and Treatment

Blood samples were collected, employing cryo-tubes with ethylenediaminetetraacetic acid, for all participants. They were centrifuged for 15 min at 1500× *g*. Plasma fraction (approximately 4 mL) was separated in a tube containing butylated 8-hydroxytoluene (BHT) (0.25 % (*w*/*v*) in ethanol) to avoid further oxidation of the sample. Then, samples were stored at −80 °C until the analysis.

Sample treatment was previously described by Peña-Bautista et al. [19]. Briefly, 5 µL of an internal standard solution (PGF2α-D4 2 µmol L-1 and D4-10-epi-10-F4t-NeuroP 1.2 µmol L-1) and 400 µL of a potassium hydroxide solution (15% *w*/*v*) were added to 400 µL of plasma to carry out the hydrolysis (40 °C, 30 min). Then, proteins were precipitated with HCl. After that, the supernatant pH was adjusted to 7. Then, samples were purified by solid phase extraction in order to preconcentrate analytes and minimize interferences. Finally, the extract was evaporated and reconstituted to be analyzed by ultra-performance liquid chromatography coupled to tandem mass spectrometry (UPLC-MS/MS) [24].

### 2.3. Statistical Analysis

Median differences between participant groups were analyzed using the chi-square test for categorical variables and the Mann–Whitney test for numerical variables. Bivariate correlations were established using the Pearson correlation. For all the analysis, significance value was *p* value < 0.05. Box-plots were used to represent the levels of isoprostanoids biomarkers.

In order to discriminate between participants groups, the elastic net logistic regression model was used to select “variables” with the glmnet package [25], due to the collinear nature and high dimensionality of the data. The elastic net regularization method of the estimated beta coefficients improves upon ordinary least squares. It linearly combines the L1 and L2 penalties of the lasso and ridge methods. Regularization parameter λ determines the amount of regularization. An optimal value for λ was determined performing a 5-fold cross-validation, which yielded the minimum cross-validated mean-squared error (CVM). A median of 500 repetitions of the cross validation was calculated in order to improve lambda´s robustness.

## 3. Results

### 3.1. Patients’ Characteristics

Demographic characteristics of the participants are described in Table 2. Participants showed median ages between 62 and 70 years old and they showed comparable normal cognitive status, with similar median RBANS.DM and CDR scores. As expected, the control group showed higher median levels of β-amyloid-42 than the preclinical group, and the control group showed lower levels of t-tau and p-tau than the preclinical group. Additionally, both groups showed similar use of medications, comorbidities and educational levels.

### 3.2. Plasma Levels of Lipid Peroxidation Lipid Compounds

**Figure 1:**
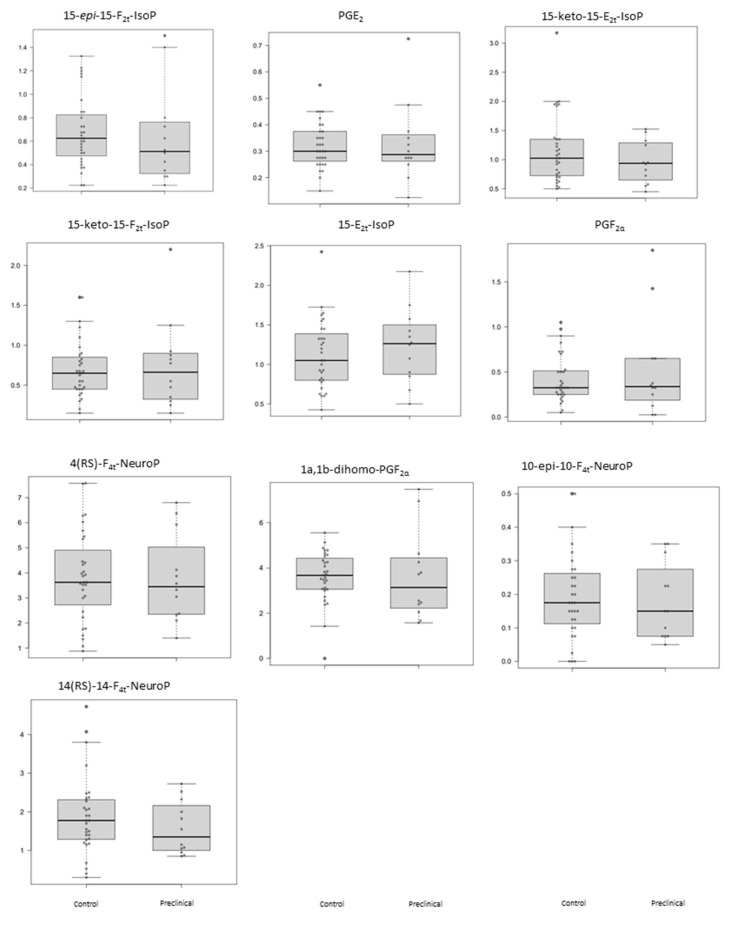
Box plots representing the concentrations in plasma samples for each analyte in control and preclinical-AD groups. Boxes represent the 1st and 3rd quartiles, and the black lines, the median.

The plasma levels obtained for the determined lipid peroxidation compounds are summarized in Table 3 for each participant group. As can be seen, these potential biomarkers did not show statistically significant differences between preclinical AD patients and healthy participants (Table 3). Figure 1 shows the corresponding boxplots, observing slight differences in median values between groups. In general, lower levels were obtained for the preclinical AD group.

**Figure 2:**
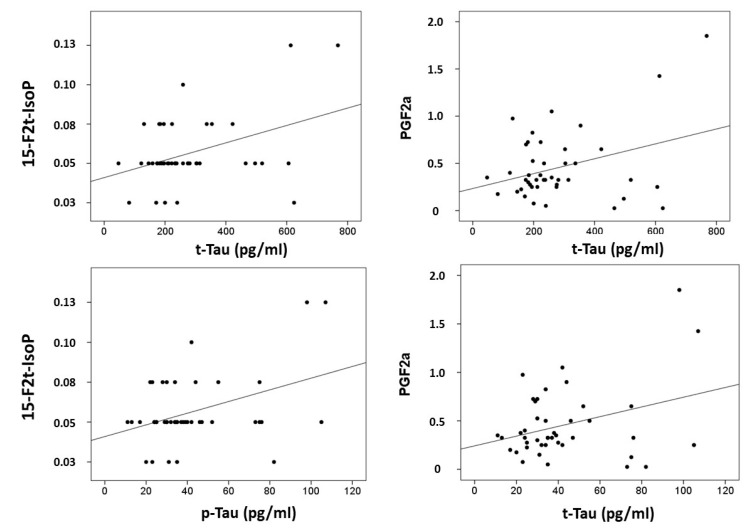
Correlation plots between plasma metabolites and CSF biomarkers.

Correlations were computed between CSF biomarkers (β-amyloid-42, tau and p-tau) and plasma lipid peroxidation biomarkers (see Figure 2). Results showed that t-tau correlated with 15-F_2t_-IsoP (*r* = 0.397, *p* = 0.008), and PGF2α (*r* = 0.339, *p* = 0.026); and p-tau correlated with 15-F_2t_-IsoP (0.401, *p* = 0.008), and PGF2α (*r* = 0.329, *p* = 0.031). In addition, correlations were assayed between neuropsychological status and plasma biomarkers. Specifically, RBANS.DM correlated with 2,3-dinor-15-*epi*-15-F_2t_-IsoP (*r* = −0.314, *p* = 0.040), 15-E_2t_-IsoP (*r* = −0.432, *p* = 0.025), 5-F_2t_-IsoP (*r* = −0.335, *p* = 0.028), 15-F_2t_-IsoP (*r* = −0.390, *p* = 0.10), and PGF2α (*r* = −0.342, *p* = 0.025). Additionally, CDR showed correlation with 15-*epi*-15-F_2t_-IsoP (*r* = 0.329, *p* = 0.031), PGE2 (*r* = 0.329, *p* = 0.031), 2,3-dinor-15-*epi*-15-F_2t_-IsoP (*r* = 0.316, *p* = 0.039), 15-keto-15-E_2t_-IsoP (*r* = 0.333, *p* = 0.029), 15-keto-15-F_2t_-IsoP (*r* = 0.319, *p* = 0.037), 15-E_2t_-IsoP (*r* = 0.363, *p* = 0.017), and 4(*RS*)-4-F_4t_-NeuroP (*r* = 0.332, *p* = 0.030).

### 3.3. Potential Diagnosis Model

**Figure 3:**
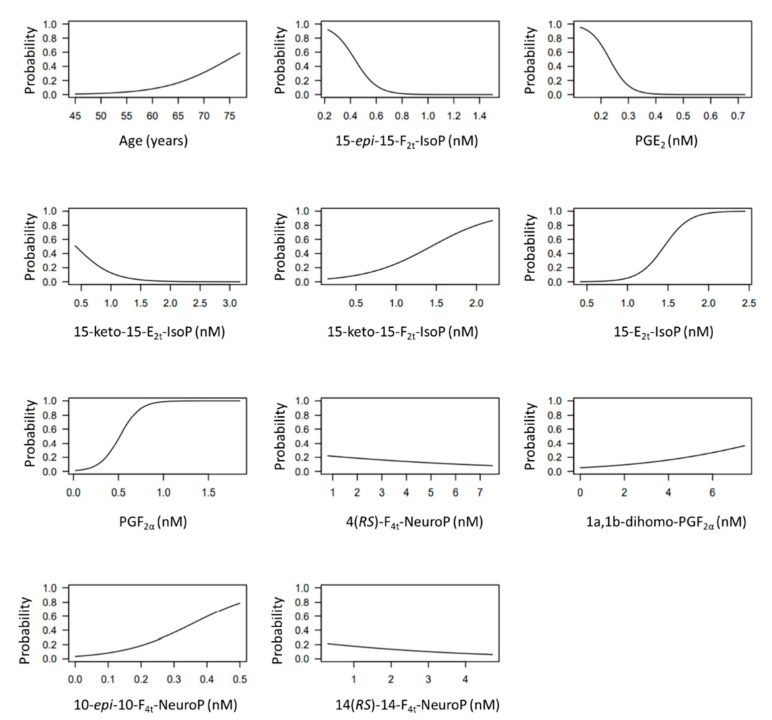
Conditional effect plots for each variable included in the model to predict the probability of preclinical-AD.

**Figure 4:**
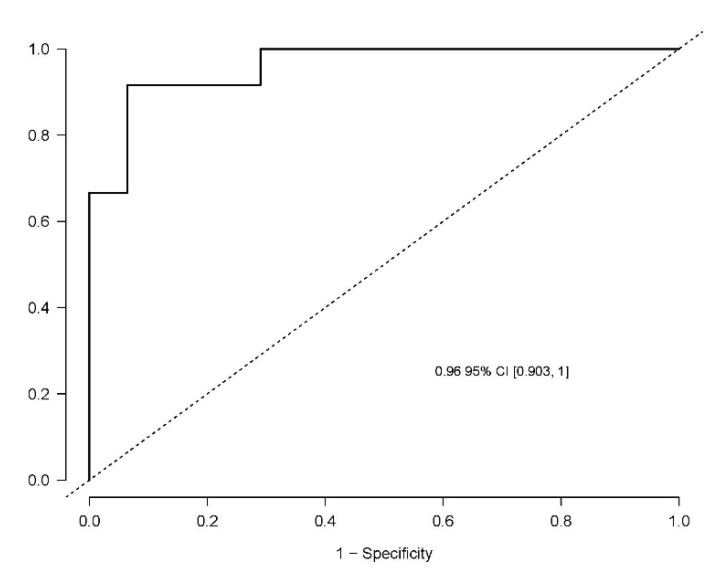
Receiver operating characteristic curve for the diagnostic model. The area under curve (AUC) is 0.96 (95% Confidence interval (CI), 0.903–1).

**Figure 5:**
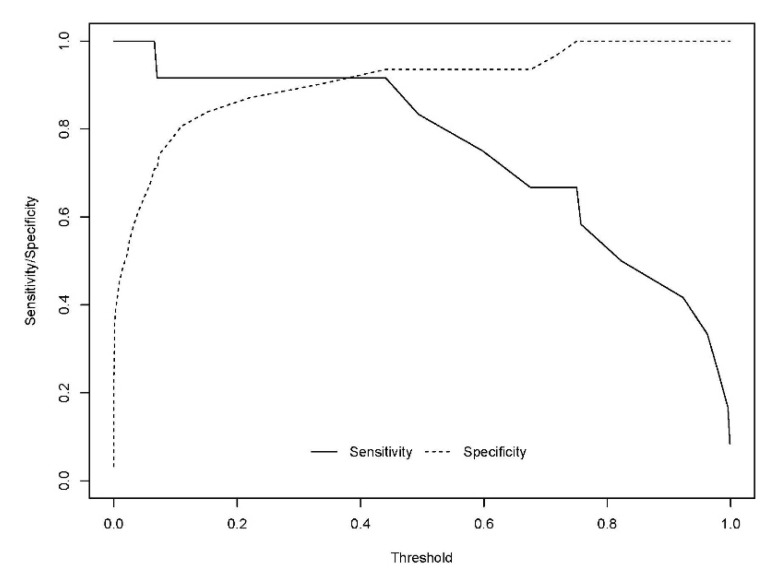
Sensitivity and specificity profile plot. The continuous line represents the relationship between the probability threshold set in the model’s prediction and the sensitivity. The dashed line represents the relationship between the probability threshold and the specificity.

The developed model included 10 analytical variables (15-*epi*-15-F_2t_-IsoP, PGE2, 15-keto-15-E_2t_-IsoP, 15-keto-15-F_2t_-IsoP, 15-E_2t_-IsoP, PGF2α, 4(*RS*)-4-F4t-NeuroP, 1a,1b-dihomo-PGF2α, 10-*epi*-10-F_4t_-NeuroP, 14(*RS*)-14-F_4t_-NeuroP), as well as age and gender. Table 4 shows the model characteristics and the tendency of the different selected biomarkers. The conditional effect for each variable is represented in Figure 3, showing the increase or decrease in preclinical-AD probability according to the levels for each variable. This model showed an AUC of 0.96 (CI 95%, 0.903–1) (Figure 4), and a validation AUC of 0.90. The sensitivity and specificity profile shows a satisfactory compromise, with high sensitivity (0.91) at a high specificity (0.93), constituting the optimum cut-off point (0.44) (Figure 5). The equation of the developed model determining the probability of suffering from preclinical-AD status is shown.

$$\mathrm{Pr}(preclinical-\mathrm{AD})=\frac{e^{LP\;}}{1+e^{\mathrm{LP}}}$$

where LP = −6.566–0.153 * Female + 0.164 *Age—11.622 * A − 28.241 * B − 3.277 * C + 2.457 * D + 6.391 * E + 8.988 * F − 0.174 * G + 0.315 * H + 9.298 * I − 0.323 * J

A: 15-*epi*-15-F_2t_-IsoP
B: PGE_2_
C: 15-keto-15-E_2t_-IsoP
D: 15-keto-15-F_2t_-IsoP
E: 15-E_2t_-IsoP
F: PGF_2__α_
G: 4(RS)-4-F_4t_-NeuroP
H: 1a,1b-dihomo-PGF_2__α_
I: 10-*epi*-10-F_4t_-NeuroP
J: 14(*RS*)-14-F_4t_-NeuroP

## 4. Discussion

In this work, some lipid peroxidation compounds were measured simultaneously in plasma samples from preclinical AD and healthy elderly participants, using UPLC-MS/MS as an analytical technique. These biomarkers did not show statistically significant different levels between both groups, although small differences could be observed for each metabolite. In addition, some of them showed a correlation with specific CSF biomarkers for AD (t-tau, p-tau) and with neuropsychological tests (RBANS.DM, CDR), showing a certain relationship with early AD development. Thus, a multivariate model was developed including some of these lipid peroxidation compounds, and showing their potential utility in discrimination between preclinical AD patients and healthy participants. In fact, the multivariate model takes into account the effect of each individual predictor, which could change in the presence of other variables, generating a composed algorithm, and it provides accurate predictions. These compounds were studied because they can reflect specific impairment of brain white matter or grey matter. However, their specificity would be determined in further studies, because there is no clear evidence that potentially detectable changes would be AD-specific, or if they would be general biomarkers of impairment of brain lipid metabolism.

In the literature, in some studies focused on searching for AD plasma biomarkers, mainly lipidic molecules were assayed [19,26]. However, most of them were based on participants with MCI and AD, all of them were patients with clinical symptoms (memory loss, cognitive decline), but none of them evaluated the group of well-characterized preclinical participants [19,27,28]. In fact, a previous work from our group was focused on the determination of lipid peroxidation compounds (isoP, NeuroP, isoF, NeuroF) in plasma samples from MCI-AD patients, developing a diagnosis model [19]. In that model, the selected compounds were 15-*epi*-15-F_2t_-IsoP, 15-E_2t_-IsoP, PGF_2α_, 4(*RS*)-F_4t_-NeuroP, 14(*RS*)-14-F_4t_-NeuroP, and Ent-7(*RS*)-7-F_2t_-dihomo-IsoP. All of them, except Ent-7(*RS*)-7-F_2t_ dihomo-IsoP, were included in the present diagnosis model to predict AD in presymptomatic stage (preclinical AD). However, higher concentrations for these compounds were found in MCI-AD patients than in healthy participants; while lower concentrations were obtained for 15-*epi*-15-F_2t_-IsoP and 4(*RS*)-F_4t_-NeuroP in preclinical AD patients. These differences could be explained by the disease progression. In addition, the new developed model included more variables (PGE_2_, 15-keto-15-E_2t_-IsoP, 15-keto-15-F_2t_-IsoP, 1a,1b-dihomo-PGF_2α_, 10-*epi*-10-F_4t_-NeuroP) in order to improve the accuracy (AUC validated = 0.90) in comparison with the previous model (AUC validated = 0.82) [19].

Recent research has focused on earlier AD stages, before the appearance of the first clinical manifestations of the disease. In general, these studies were about plasma β-amyloid-42/β-amyloid-40 ratio, showing an AUC of 0.78 in the discrimination between normal cognitive individuals with PET β-amyloid positivity and negativity [29]. In addition, plasma β-amyloid levels showed an association with dementia (determined by Mini Mental State Examination (MMSE) and the Geriatric Mental State Schedule (GMS)) and AD [30]. However, other study showed that plasma β-amyloid levels could not predict AD in preclinical participants [31]. A further study focused on plasma p-tau revealed its utility in AD diagnosis and prognosis, showing increased values since preclinical stages and an accuracy of 85% in AD dementia diagnosis [32]. However, the present work is the first study evaluating lipid peroxidation compounds in preclinical AD patients accurately diagnosed by CSF biomarkers.

Similarly, some of the studied biomarkers were lipidic compounds in plasma from preclinical AD participants [33]. In fact, the study carried out by Mapstone et al. analyzed lipids (phosphatidylcholine, Lysophosphatidylcholine, acylcarnitines, etc.), and it was carried out following the progression along 5 years, showing their potential utility as progression AD biomarkers [28].

The model developed in the present work was based on the plasma levels of 10 lipid peroxidation compounds. It is shown that an increase in the levels of these biomarkers (15-keto-15-F_2t_-IsoP, 15-E_2t_-IsoP, PGF_2α_, 10-*epi*-10-F_4t_-NeuroP) could increase the probability of suffering from AD. Previous studies showed the utility of models based on plasma lipids as predictor approach of conversion amnestic MCI to AD or AD progression since preclinical stages [9,28]. The biomarkers determined in these studies are mainly related to membrane integrity, while ours are derived from oxidative stress. Another panel including 17 lipids can predict cognitive decline and brain atrophy in AD and it is related to clinical diagnosis in AD and t-tau CSF levels [34].

Early AD diagnosis remains a big challenge for human sciences. There is a high need for easily available biomarkers now that specific biomarkers have been described. These specific biomarkers are invasive and expensive; so minimally invasive biomarkers are in demand. The utility of these putative biomarkers can be found in the diagnostic paradigm, identifying people at risk for developing cognitive impairment, with a biological suspicion of specific or non-specific neurodegeneration, or other pre-diagnostic characteristics. In addition, these biomarkers could be useful in identifying subgroups with different disease evolution, different therapeutic response, and different neuropsychological dysfunction.

Among the study limitations, it is important to highlight the small sample used. This limitation is an evident issue and the results of a study with a higher number of cases cannot be anticipated. However, the present study could be considered exploratory. It is important to remark that the participants were selected in an asymptomatic stage, and highlight the difficulties of realizing CSF studies in asymptomatic cases. Another limitation is the exclusion of cases with other similar neurodegenerative diseases. Different patterns of biomarkers are expected in other neurodegenerative diseases, but in the present study, they were not evaluated. Therefore, these are preliminary results and further analysis in a large external cohort is required.

## 5. Conclusions

Lipid peroxidation biomarkers were determined in plasma from participants with preclinical AD and healthy elderly participants, showing no differences individually. However, these biomarkers showed a correlation with other specific AD CSF biomarkers and neuropsychological status. The multivariate model including 10 of these biomarkers constitutes a promising diagnostic tool to be applied to the general population in early AD detection. However, further validation studies are necessary to confirm the utility of this potential model for preclinical AD diagnosis.

## Figures and Tables

**Table 1:** Clinical assessment to classify the study participants.

Clinical Assessment	Classification of Participants	
	Control	Preclinical AD
RBANS.DM ^1^	≥85	≥85
FAQ ^2^	<9	<9
CDR ^3^	0-0.5	0–0.5
MMSE ^4^	≥27	≥27
CSF t-tau (pg mL^−1^)	<485	>485
CSF p-tau (pg mL^−1^)	<56	>56
CSF β-amyloid42 (pg mL^−1^)	>725	<725
CSF t-tau/β-amyloid42	<0.51	>0.51

^1^ RBANS.DM, repeatable battery for the assessment of neuropsychological status-delayed memory (standard score; cut-off point < 85). ^2^ FAQ, functional activities questionnaire (direct score; cut-off point > 9). ^3^ CDR, clinical dementia rating, values: 0, 0.5, 1, 2. ^4^ MMSE, mini-mental state (cut off point < 27). CSF, cerebrospinal fluid.

**Table 2:** Participants’ clinical and demographic description.

Variable		Control Group (*n* = 31) Median (1st, 3rd Quartile)	Preclinical Group (*n* = 12) Median (1st, 3rd Quartile)
Age (years)		62 (58.5, 67)	70 (60.75, 74)
Gender (Female, *n* (%))		19 (61.29%)	6 (50%)
Smoke (Yes, *n* (%))		6 (27.27%)	1 (14.29%)
Alcohol (Yes, *n* (%))		6 (27.27%)	0 (0%)
RBANS.DM (score)		98 (94, 102)	94.5 (87, 100.25)
RBANS.A (score)		91 (82, 98.5)	85 (78, 91)
RBANS.L (score)		90 (83, 94)	88.5 (82.5, 94.25)
RBANS.VC (score)		92 (84, 105)	87 (75, 105)
RBANS.IM (score)		87 (83, 98.5)	85 (81.75, 94)
CDR (score)		0.5 (0, 0.5)	0.5 (0, 0.5)
CSF β-amyloid-42 (pg mL^−1^)		1224 (975.5, 1409.5)	571.5 (407, 683.29)
CSF t-tau (pg mL^−1^)		212 (181.5, 259)	443.5 (256.75, 607.75)
CSF p-tau (pg mL^−1^)		34 (26.5, 38.5)	74 (40.75, 86)
CSF t-tau/β-amyloid-42		0.18 (0.16–0.21)	0.70 (0.51–0.97)
FAQ (score)		1 (0, 3.5)	1 (0, 3)
GDS (score)		11 (5.5, 13)	5 (3.75, 9)
Educational level	Basic/primary	10 (32.26%)	4 (33.33%)
	Secondary	7 (22.58%)	2 (16.67%)
	Universitary	14 (45.16%)	6 (50%)
Medication (*n*, (%))			
Statins		9 (40.91%)	3 (42.86%)
Fibrates		0 (0%)	1 (14.29%)
Morphics		0 (0%)	0 (0%)
ACEI		1 (4.55%)	0 (0%)
Neuroleptics		2 (9.09%)	0 (0%)
Benzodiazepines		6 (27.27%)	2 (28.57%)
Antiepileptics		1 (4.55%)	0 (0%)
Anticoagulants		0 (0%)	0 (0%)
Antihipertensives		7 (31.82%)	2 (28.57%)
Corticoids		1 (4.55%)	0 (0%)
Anti-inflammatory		3 (13.64%)	0 (0%)
Comorbidity (*n*, (%))			
Dyslipidemia		11 (50%)	3 (42.86%)
Diabetes		9 (40.91%)	1 (14.29%)
Hypertension		8 (36.36%)	2 (28.57%)
Heart Disease		1 (4.55%)	0 (0%)
Cerebrovascular		1 (4.55%)	0 (0%)
Depression		4 (18.18%)	2 (28.57%)
Anxiety		3 (13.64%)	2 (28.57%)

RBANS, Repeatable Battery for the Assessment of Neuropsychological Status (DM, delayed memory; A, attention; L, learning; VC, visuospatial/constructional; IM, immediate memory); CDR, clinical dementia rating; CSF cerebrospinal fluid; FAQ, functional activities questionnaire; GDS, geriatric depression scale; ACEI, acetylcholinesterase inhibitors.

**Table 3:** Plasma levels of lipid peroxidation compounds.

Variable (nmol L^−1^)	Control (*n* = 31)	Preclinical (*n* = 12)	*p* Value
	Median (1st, 3rd Quartile)	Median (1st, 3rd Quartile)	
15-*epi*-15-F_2t_-IsoP	0.62 (0.48, 0.82)	0.51 (0.34, 0.74)	0.414
PGE_2_	0.3 (0.26, 0.38)	0.29 (0.27, 0.36)	0.738
2,3-dinor-15-*epi*-15-F_2t_-IsoP	0.03 (0, 0.03)	0.03 (0.02, 0.03)	0.602
15-keto-15-E_2t_-IsoP	1.02 (0.72, 1.35)	0.94 (0.69, 1.27)	0.384
15-keto-15-F_2t_-IsoP	0.65 (0.45, 0.85)	0.66 (0.34, 0.89)	0.926
15-E_2t_-IsoP	1.05 (0.8, 1.39)	1.26 (0.89, 1.46)	0.478
5-F_2t_-IsoP	2.75 (2.16, 3.19)	2.35 (1.63, 2.9)	0.414
15-F_2t_-IsoP	0.05 (0.05, 0.05)	0.05 (0.05, 0.07)	0.430
PGF_2α_	0.32 (0.25, 0.51)	0.34 (0.22, 0.65)	0.968
4(*RS*)-4-F_4t_-NeuroP	3.62 (2.72, 4.9)	3.45 (2.36, 4.58)	0.800
1a,1b-dihomo-PGF_2α_	3.67 (3.06, 4.43)	3.14 (2.31, 4.34)	0.478
10-*epi*-10-F_4t_-NeuroP	0.17 (0.11, 0.26)	0.15 (0.07, 0.25)	0.698
14(*RS*)-14-F_4t_-NeuroP	1.77 (1.29, 2.31)	1.35 (1.03, 2.08)	0.355
*ent*-7(*RS*)-7-F_2t_-dihomo-IsoP	0 (0, 0)	0 (0, 0.01)	0.414
17-F_2t_-dihomo-IsoP	0 (0, 0)	0 (0, 0)	1.000
17-*epi*-17-F_2t_-dihomo-IsoP	0 (0, 0)	0 (0, 0)	1.000
17(*RS*)-10-*epi*-SC-Δ^15^-11-dihomo-IsoF	0 (0, 0)	0 (0, 0)	0.679
7(*RS*)-ST-Δ^8^-11-dihomo-IsoF	0 (0, 0.22)	0 (0, 0)	0.165
Neurofurans	0.27 (0.19, 0.37)	0.24 (0.21, 0.41)	0.679
Isofurans	0.52 (0.4, 0.65)	0.5 (0.41, 0.69)	0.718
Dihomo-isoprostanes	0.15 (0.14, 0.17)	0.15 (0.13, 0.17)	0.883
Dihomo-isofurans	0.01 (0.01, 0.02)	0.01 (0.01, 0.02)	0.883
Neuroprostanes	0.64 (0.49, 0.76)	0.59 (0.45, 0.77)	0.679
Isoprostanes	1.5 (1.25, 1.84)	1.32 (1.14, 1.67)	0.328

**Table 4:** Model parameters.

Variables	Estimate	Exponential Estimate. (e ^Estimate^)
(Intercept)	−6.566	0.001
Gender (Females)	−0.153	0.858
Age	0.164	1.178
15-epi-15-F_2t_-IsoP	−11.622	0
PGE_2_	−28.241	0
15-keto-15-E_2t_-IsoP	−3.277	0.038
15-keto-15-F_2t_-IsoP	2.457	11.671
15-E_2t_-IsoP	6.391	596.158
PGF_2α_	8.988	8003.721
4(RS)-4-F_4t_-NeuroP	−0.174	0.841
1a,1b-dihomo-PGF_2α_	0.315	1.371
10-*epi*-10-F_4t_-NeuroP	9.289	10,823.421
14(*RS*)-14-F_4t_-NeuroP	−0.323	0.724
Lambda	0.004	

## Data Availability

The data presented in this study are available on request from the corresponding author.
